# Preparation and Performance of Poly(butyl fumarate)-Based Material for Potential Application in LED Encapsulation

**DOI:** 10.3390/ma10020149

**Published:** 2017-02-11

**Authors:** Liang Wang, Da-Gang Guo

**Affiliations:** State Key Laboratory for Mechanical Behavior of Materials, School of Materials Science and Engineering, Xi’an Jiaotong University, Xi’an 710049, China; wanglcody@163.com

**Keywords:** poly(butyl fumarate)-based polymer, composites, characterization, UV-curing, LED encapsulant

## Abstract

A UV-curable poly(butyl fumarate) (PBF)/poly(propylene fumarate)-diacrylate (PPF-DA) hybrid material with good performance for LED encapsulation is introduced in the paper. They have been prepared by radical polymerization using PBF and PPF-DA macromers with a UV curing system. PBF and PPF-DA were characterized by Fourier-transform infrared (FT-IR) and H-nuclear magnetic resonance (^1^H NMR). The thermal behavior, optical and mechanical properties of the material were examined by thermogravimetric analysis (TGA), differential scanning calorimetry (DSC), ultraviolet-visible spectroscopy (UV–vis), and a material testing system mechanical testing machine, respectively. The results indicated that the hybrid material has a suitable refractive index (*n* = 1.537) and high transmittance (99.64% in visible range) before/after thermal aging. With the increasing of the double bond ratio from 0.5 to 2, the water absorption ratios of the prepared encapsulation material were 1.22%, 1.87% and 2.88%, respectively. The mechanical property experiments showed that bonding strength was in the range of 1.86–3.40 MPa, tensile-shear strength ranged from 0.84 MPa to 1.57 MPa, and compression strength was in the range of 5.10–27.65 MPa. The cured PBF/PPF-DA hybrid material can be used as a light-emitting diode (LED) encapsulant, owing to its suitable refractive index, high transparency, excellent thermal stability, lower water absorption, and good mechanical properties.

## 1. Introduction

In recent years, light-emitting diodes (LEDs) have increasingly been used for a variety of lighting applications [1,2,3]. They have attracted great interest because of their advantages in terms of high efficiency, low power consumption, environmental benefits, among other factors [4,5,6]. With the rapid development of LED technology, some stringent requirements for the high-performance encapsulants have to be satisfied [7,8,9]. Therefore, encapsulants should have a performance of high transparency in the UV–visible region, a suitable refractive index, appropriate mechanical strength, excellent thermal stability, good dimensional stability, and well-established operation process. Owing to inexpensive and good adhesive properties, epoxy resins have been the standard choice for encapsulation of indicator LEDs [10,11,12]. However, they are rather brittle, hydrophilic, and easily suffer from yellowing during thermal or UV curing processes [2,13,14]. Silicone-based materials have the properties of good thermooptical stability and wide range of service temperatures, but they have very poor adhesion and mechanical properties [15,16]. Phenyl-siloxane hybrid materials show remarkable thermal resistance against yellowing, compared with silicone encapsulants. Nevertheless, they have low optical transparency [3]. Acryl or epoxy polymers have low thermal resistance, due to the absence of siloxane bonds. Thus, they are not appropriate for use as UV-curable LED encapsulants [17]. Zhao et al. (2014) observed that the novel inorganic–organic siloxane hybrid material was examined to confirm its feasibility for use as an encapsulant for LEDs [1]. Yang et al. (2010) found that inorganic–organic nanohybrid materials (hybrimers) are a candidate for use as an LED encapsulant due to their good transparency and high luminescence efficiency [5]. 

A newly generated LED encapsulant with the abovementioned advantages has been proposed and thus further opened new fields for polymer synthesis [18,19]. It is interesting to develop polymers with integrated functions based on molecular design and polymer synthesis. Poly(butyl fumarate)-based polymers are attractive materials for applications in many fields. Nevertheless, to date, according to the literatures, there are few reports on using poly(butyl fumarate)-based polymer for LED encapsulation. Poly(butyl fumarate) (PBF) is a linear polyester based upon fumaric acid. The fumarate double bond in PBF can be crosslinked with various reagents to form polymer networks [20,21,22]. Compared with poly(propylene fumarate), PBF has low viscosity, making handling of the polymer somewhat convenient [23]. Similarly, poly(propylene fumarate)-diacrylate (PPF-DA) is based upon the same repeat unit as PBF, containing ester groups. The crosslinking reaction can be carried out at a defect site using bis(2,4,6-trimethylbenzoyl) phenylphosphine oxide [22,23], indicating that the acrylate bond participates more in the formation of the PBF/PPF-DA polymer networks. This increase is attributed to the greater reactivity of the acrylates, as well as the biased affinity of both groups toward the acrylate bond. The behavior has been observed with similar fumarate–acrylate radical copolymerizations [24]. The polymer networks have demonstrated low water absorption and high strength [25]. Many of the organic polymer materials being used in light-emitting devices are strongly anisotropic [26]. Measurements of optical anisotropy provide direct information on the orientation of polymer molecules in the crystalline and amorphous regions. Owing to high transmittance, the refractive index and birefringence measurements are quite suitable for polymer materials. They were determined without correlation for biaxially stretched poly(butyl fumarate)-based polymer films at a microwave frequency [27]. Birefringence is the optical property of a material having a refractive index that depends on the polarization and propagation direction of light. These optically anisotropic materials are said to be birefringent. The birefringence is often quantified as the maximum difference between refractive indices exhibited by the material. Normally, the refractive indices are measured at discrete wavelengths for the ordinary and extraordinary, and their differences are calculated as the birefringence [28]. Our recent research results show this series of polymer networks have advantages over other materials owing to their suitable refractive index, high transparency, appropriate mechanical strength, excellent thermal stability, good dimensional stability, and well-established operation process [23].

Based on these, we attempted the design and synthesis of different double-bond-ratio PBF/PPF-DA polymer networks which exhibit a suitable refractive index, wide wavelength range transparency, excellent thermal stability, low viscosity prior to curing, and good mechanical properties. Presented in this work is as an example of development of multifunctional polymers. Current results indicate that PBF/PPF-DA polymer networks are potential encapsulating materials for light-emitting diode devices.

## 2. Experiments

### 2.1. Materials

Starting materials such as diethyl fumarate, 1,4-butanediol, zinc chloride, hydroquinone, and bis(2,4,6-trimethylbenzoyl) phenylphosphine oxide were purchased from Aladdin Chemistry Co., Ltd., (Shanghai, China) and Beijing J&K Scientific Ltd., (Beijing, China). All other reagents and solvents were reagent grade and used without further purification, except methylene chloride, which was purified by standard methods. Regenerating molecular sieve absorbents were used for methylene chloride dehydration.

### 2.2. Preparation of LED Encapsulation Material

**Scheme 1:**
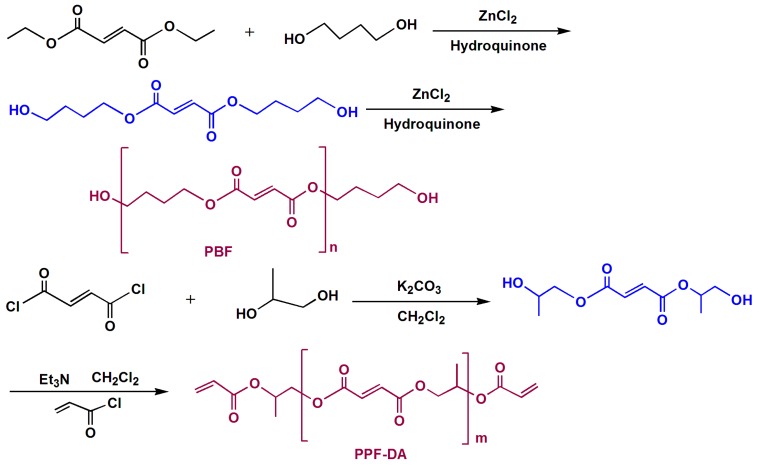
Synthesis of poly(butyl fumarate) (PBF) and poly(propylene fumarate)-diacrylate (PPF-DA).

Poly(butyl fumarate) (PBF) was synthesized following a two-step procedure (Scheme 1) [22,23,29]. First, the intermediate product bis(hydroxybutyl) fumarate was synthesized. Diethyl fumarate (250 mmol, 430 mg) and 750 mmol (676 mg) of 1,4-butanediol were reacted using 2.5 mmol (3.4 mg) of zinc chloride as a catalyst and 0.5 mmol (0.6 mg) of hydroquinone as a radical inhibitor. In this step, the reaction was performed in a heated vessel under mechanical stirring, with a gradual increase in temperature from 110 to 140 °C. The reaction was run for approximately 8 h under a nitrogen blanket, producing bis(hydroxybutyl) fumarate as the main product and ethanol as a byproduct. The bis(hydroxybutyl) fumarate was then transesterified, producing poly(butyl fumarate). The synthesis reaction underwent continuous heating for 7 h at a temperature of 130 °C and a low pressure of 5 mmHg. This product was dissolved in methylene chloride to allow for purification. PBF was first washed with acid (1.85 vol % HCl in H_2_O) to remove the zinc chloride, and then purified with two washes each of both pure water and brine. The organic phase was dried with sodium sulfate. The concentrated product was then precipitated in petroleum ether twice to remove the radical inhibitor. Finally, all solvents were removed from the PBF by rotary evaporation followed by vacuum-drying. Selected IR (KBr pellet, cm^−1^): *ν* (–OH) 3432; *ν* (C–H) 2950; *ν* (C=O) 1724; *ν* (C=C) 1647; *ν* (–CH, –CH_2_, –CH_3_) 1465; *ν* (–C=CH–) 980. ^1^H NMR (400 MHz, CDCl_3_): δ 6.85 (s, 2H, –CH=CH–), 4.27~4.22 (m, 2H, OH), 3.69 (s, 4H, CH_2_), 2.08 (s, 4H, CH_2_), 1.72 (dq, *J* = 55.2, 3.6 Hz, 4H, CH_2_), 1.32 (t, *J* = 7.2 Hz, 4H, CH_2_).

Scheme 1 shows the synthetic route for the preparation of poly(propylene fumarate)-diacrylate (PPF-DA). PPF-DA was synthesized in two reactions as previously described. The details of the experimental (simulation) procedure has already been published elsewhere [22]. Fumaryl chloride (306 mg, 200 mmol), 1,2-propanediol (457 mg, 600 mmol), anhydrous potassium carbonate (415 mg, 300 mmol), bis(hydroxypropyl) fumarate (174 mg, 75 mmol), triethylamine (228 mg, 225 mmol), acryloyl chloride (133 mg, 147 mmol). Selected IR (KBr pellet, cm^−1^): *ν* (C–H) 2980; *ν* (C=O) 1725; *ν* (–CH, –CH_2_, –CH_3_) 1455; *ν* (C–O–C) 1154; *ν* (–C=CH–) 978. ^1^H NMR (400 MHz, CDCl_3_): δ 6.88 (bs, 2H, –CH=CH–), 6.43 (dd, *J* = 17.2 Hz, 1H, –CH=CH_2_), 6.17~6.09 (m, 1H, –CH=CH_2_), 5.89~5.86 (m, 1H, –CH=CH_2_), 5.33~5.27 (m, 1H, CH), 4.36~4.20 (m, 2H, CH_2_), 1.36~1.33 (m, 3H, CH_3_).

**Scheme 2:**
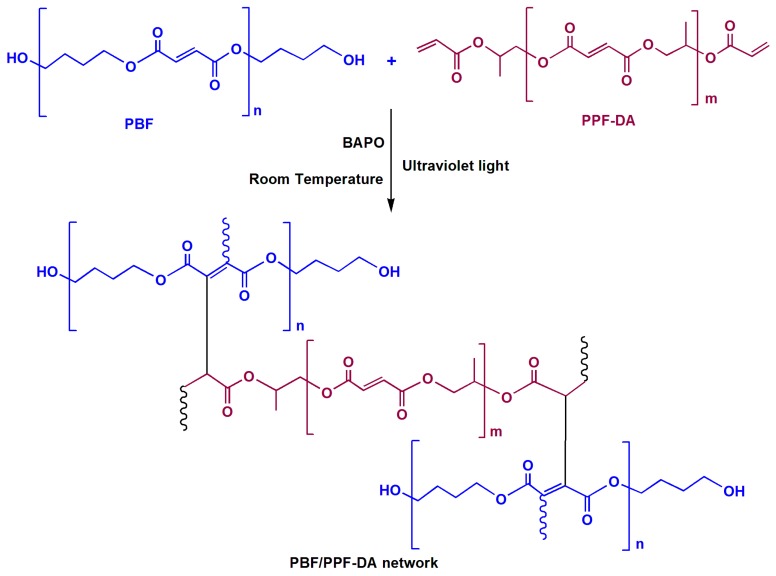
Synthesis of PBF/PPF-DA polymer networks.

Polymer mixtures of PBF and PPF-DA, as dictated by the double-bond ratio, were prepared by dissolving the appropriate amounts of each component in methylene chloride. The two solutions were combined, stirred for 40 min, and vacuum-dried to remove all solvents. The BAPO (4-Benzylideneamino-2,2,6,6-tetramethylpiperidine-1-oxyl) was administered to 1 g of PBF/PPF-DA blend in 0.03 mL of a methylene chloride solution (1 g BAPO per 10 mL methylene chloride) so that the initiator concentration was 0.3 wt %. Three formulations of PBF/PPF-DA networks of double-bond ratios 0.5, 1 and 2 were used in the study. The double-bond ratio is defined as the ratio of acrylates in PPF-DA to fumarates in PBF. For specimen preparation, the crosslinking mixture was placed into a rectangle mold (length × width × thickness = 10 mm × 10 mm × 1 mm). Similarly, the paste was thoroughly mixed with a vortexer and then poured in a cylindrical glass vial (4 mm diameter, 10 mm length). The molds were positioned roughly 10 cm below four bulbs that provided the majority of light at 365 nm and an intensity of approximately 300 mw·cm^−2^ within 4 min. The synthetic route for the preparation of LED encapsulation material is shown in Scheme 2.

### 2.3. Degree of Conversion

The degree of UV-cured material with a double-bond ratio of 1 was determined by using a Nicolet nexus Fourier-transform infrared (FT-IR) spectrometer. In order to monitor the curing behavior and for further characterization of the coatings, the UV-cured films, with the thickness of 50 μm, were prepared by coating such mixtures on the polyethylene terephthalate (PET) film. Then, they were irradiated with ultraviolet light within 4 min using a light box (300 mw·cm^−2^, 365 nm, OmniCure series 2000).

### 2.4. Measurements and Characterization

#### 2.4.1. FT-IR and ^1^H NMR 

FT-IR spectra of the synthesized polymers were recorded between 4000 and 400 cm^−1^ with a Nicolet Nexus FT-IR spectrometer. ^1^H NMR spectra were performed on a Bruker-400 spectrometer with CDCl_3_ as solvent and tetramethylsilane (TMS) as the internal reference.

#### 2.4.2. TGA and DSC

Thermogravimetric analyses (TGA) were carried out by a PerkinElmer Pyris-1 analyzer (TG209C, Netzsch, Germany) to investigate the thermal stability of UV-cured PBF/PPF-DA composite materials. In the present study, the cured samples were cut into small pieces, and about 10 mg sample was taken and heated in a dry nitrogen atmosphere from 25 to 600 °C at the heating rate of 10 °C·min^−1^ in all cases. Different scanning calorimeter (DSC) measurements were performed on a Netzsch DSC 200 pc thermal analysis apparatus in a nitrogen atmosphere. All the specimens were first heated to 400 °C and maintained at 400 °C for 5 min to remove the influence of background, and then quenched to 25 °C, followed by heating from 25 to 400 °C with a heating rate of 5 °C·min^−1^. The glass-transition temperature (Tg) values were recorded during the second heating scan and taken as the midpoint of the heat capacity change.

#### 2.4.3. Optical Properties

UV–visible spectrometer (UV-3600, Shimadzu, Kyoto, Japan) was used to examine the transmittance of the UV-cured PBF/PPF-DA composite materials. Both the bare PET for the control and the sample, which was coated on the PET film at 2 mm thickness, were set on the instrument. The PBF/PPF-DA blends were irradiated with ultraviolet light within 4 min using a light box (300 mw·cm^−2^, 365 nm, OmniCure series 2000) under an air condition to obtain samples with a substantial thickness of 1 mm. The transmittance was determined in the visible range of 380~800 nm. The refractive index of the sample coated in a PET film was detected using spectroscopic ellipsometry (PZ-2000, Jobin Yvon S.A.S, France) at a wavelength of 557 nm. Thermal aging studies were conducted in an electric constant-temperature drying oven.

#### 2.4.4. Water Absorption Test

The water uptake of UV-curable PBF/PPF-DA material was evaluated by soaking the sample in distilled water and measuring the weight periodically. Before the experiments, the samples were dried in a vacuum oven at about 40 °C for 72 h to stabilize the weight. Then, the samples with average dimension of 4 mm (diameter) × 10 mm (length) were immersed in boiling distilled water. The water absorption ratio of the sample was calculated as the following equation, where *m*_1_ and *m*_2_ are the masses of wet and dry samples, respectively.

$$Water\;absorption\;ratio=\frac{m_{1}-m_{2}}{m_{2}}\times 100\%$$

#### 2.4.5. Mechanical Properties

The strengths (tensile-shear strength, bonding strength, and compression strength) of the cured specimens were measured with a material testing system mechanical testing machine (electro-hydraulic servo universal testing machine PLD-5kN). Tensile-shear strength testing was carried out in accordance to the Adhesives-determination of tensile shear strength of rigid to rigid bonded assemblies (GB/T 7124-2008/ISO 4587: 2003). Bonding strength testing was performed in accordance to Adhesives-determination of tensile strength of butt joint (GB/T 6329-1996 eqv ISO 6922: 1987). Compression strength testing was carried out in accordance to GB/T 1041-92. The reported results of tensile and impact testing were averages of at least five to seven measurements.

## 3. Results and Discussion

### 3.1. Characterization of Chemical Modifications

A number of synthetic techniques for poly(propylene fumarate) have been reported [30,31,32]. Similarly, the common method for synthesizing poly(butyl fumarate) follows a two-step procedure, beginning with diethyl fumarate and 1,4-butanediol, and involving bis(hydroxybutyl) fumarate as an intermediate (Scheme 2). The synthesis of PPF-DA and PBF were similar. PPF-DA was prepared by the method described by Wang et al. [23], with minor modification. These polymers were characterized by IR spectra and ^1^H NMR.

**Figure 1:**
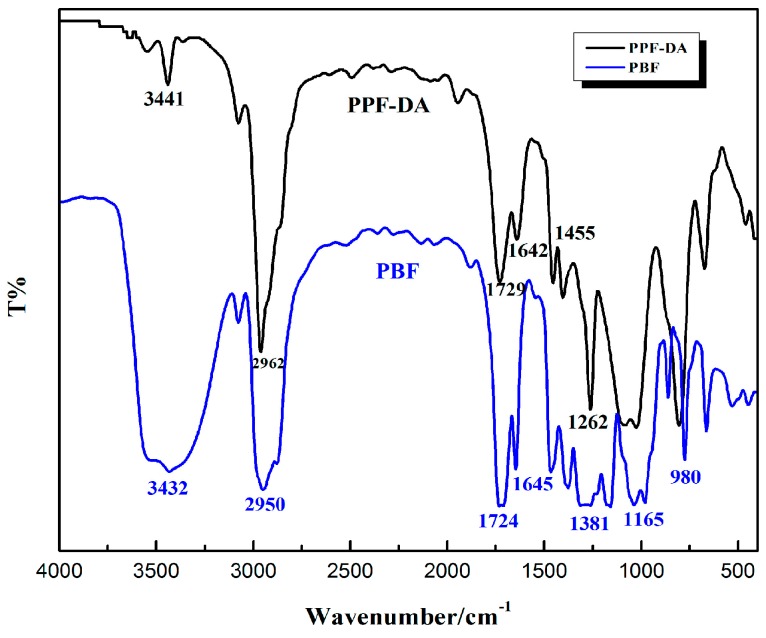
Fourier-transform infrared (FT-IR) spectra of PBF and PPF-DA are presented in 4000–400 cm^−1^ range.

Figure 1 shows that the polymers had similar absorption peaks in the FT-IR spectra. Wavenumbers of the selected vibration of IR spectra of the polymers are listed in Figure 1 of the experimental section. The absorbance at 1729, 1642, 1455, 1262, and 978 cm^−1^ are the typical carbonyl stretching, carbon–carbon double bond, methylene scissoring (methyl asymmetric) bend, C–O stretch, and C–H bend, respectively, indicating the successful synthesis of PPF-DA. Further evidence for reaction at both ends came from the FT-IR spectrum of PPF-DA, which showed no OH stretching band appeared in the region of 3500–3100 cm^−1^. IR spectra were especially valuable for the characterization of PBF. Similarly, the existence of the IR peak at 3432 cm^−1^ for a broad OH stretch centered. The absorption bands at 1724 and 1645 cm^−1^ are the ester carbonyl and C=C stretch. After transesterification of the intermediate, a noticeable decrease of the OH band at 3432 cm^−1^ was observed because of the removal of end 1,4-butanediol. The change of IR spectra strongly supported the progress of transesterification. The spectra also corroborate previous characterizations of the PBF polymeric.

**Figure 2:**
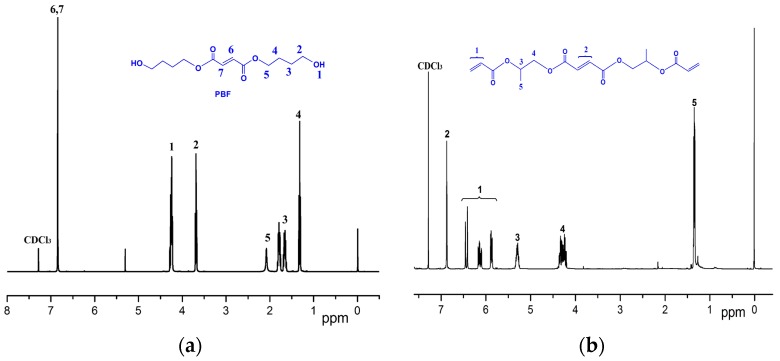
^1^H NMR spectrum of PBF (**a**) and PPF-DA (**b**).

The ^1^H-NMR spectrum of the polymers is shown in Figure 2. ^1^H NMR spectra data for the polymers are given in experimental, respectively. Chemical shift values of the polymers are in reasonable agreement with literature data for the similar compounds [25]. In Figure 2a, seven multiplets were observed. Similarly, six multiplets were observed in Figure 2b. The signals of the olefinic protons can be clearly identified at 6.85 and 6.88 ppm. The signals at chemical shifts of 1.36–1.32 ppm can be assigned to the methylene protons. The characteristic signals located at 5.33–5.27 ppm and 4.36–4.20 ppm in the ^1^H NMR spectrum can be attributed to the methine and methylene protons of the propyl diol. It was found from the ^1^H NMR spectrum of PPF-DA that the integration ratio of acryl protons to fumarate protons was 3 to 1. This ratio suggested that both terminal hydroxyl groups in di-(2-hydroxypropyl) fumarate were derivatized with an acrylate group. Difference in the chemical shift might be attributed to the formation of intermolecular interactions with solvent. 

### 3.2 Curing Behavior Monitored by FT-IR

**Figure 3:**
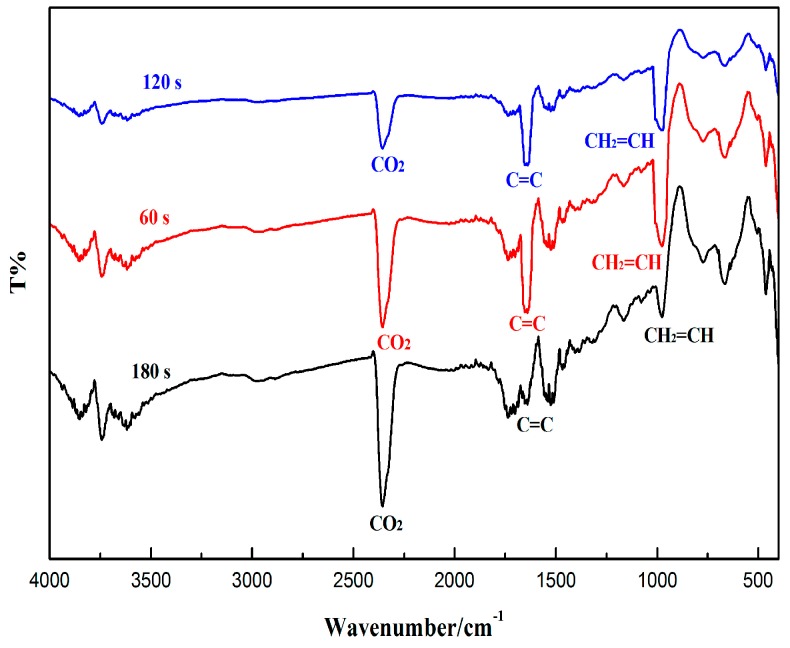
FT-IR spectra of PBF/PPF-DA showing the decrease in intensity of absorption bands at 1646 cm^−1^ and 974 cm^−1^ as a function of irradiation time.

The conversions for the photopolymerization reactions were evaluated on the basis of their FT-IR spectra. Figure 3 shows FT-IR spectra of PBF/PPF-DA UV-curable coatings as a function of time with a double-bond ratio of 1. It was found that the absorption band of acrylate group (C=C) at 1646 and 974 cm^−1^ decreased with increasing UV-curing time because C=C bonds in the reactive monomer took part in the crosslinking reaction as the UV-curing process [33,34]. This indicates that the degree of completion of the curing reaction was increased with increasing ultraviolet illumination time.

### 3.3. Thermal Behavior Analysis

**Figure 4:**
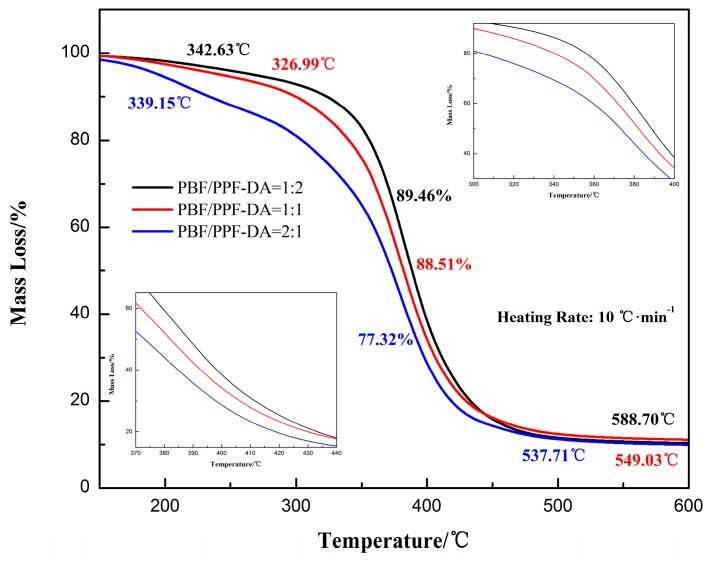
Thermogravimetric analysis (TGA) plot curves of the PBF/PPF-DA polymer networks with double-bond ratios of 0.5, 1 and 2.

**Figure 5:**
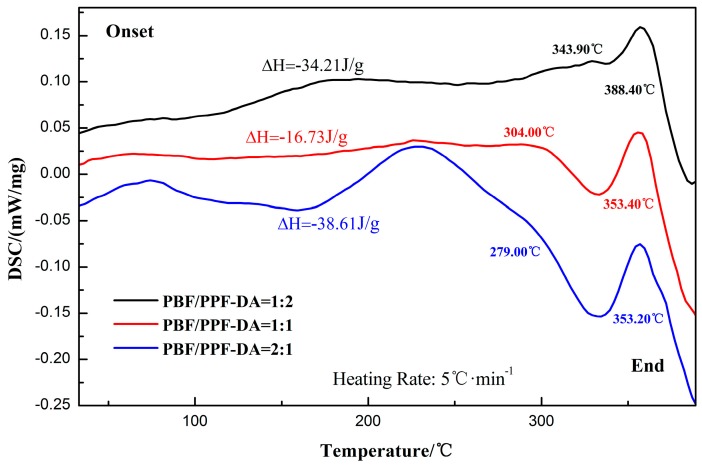
Differential scanning calorimetry (DSC) heating curves for the PBF/PPF-DA polymer networks with double-bond ratios 0.5, 1 and 2.

Thermal stability of the encapsulation material is an important factor for LED application, which is generally evaluated by differential scanning calorimetry (DSC) and thermal gravimetric analysis (TGA) techniques. The TGA traces for cured polymers are included in Figure 4. The experimental weight loss values are in good agreement with the calculated values. The DSC curves of encapsulation material and values of glass transition temperatures (Tgs) from DSC are shown in Figure 5.

Figure 4 represents the TGA curves of PBF/PPF-DA networks. There is one obvious weight loss stage in the TGA curves. All samples have a one-stage decomposition process, and a rapid weight loss is observed at around 374.01~383.58 °C. Observed from Figure 4, PBF/PPF-DA polymer networks with a double-bond ratio of 0.5 keeps thermal stability up to 342.63 °C without any weight loss, which means the compound could retain structural integrity above this temperature. The performance is slightly higher than that previously reported in the literature [23]. The composite material undergoes one major stage of weight loss between 342.63 and 588.70 °C, and the endothermic peak is in the temperature of 383.58 °C. The total weight loss at this stage closes at 89.46%. One obvious weight loss of PBF/PPF-DA networks with a double-bond ratio of 1 can be clearly identified, which starts from 326.99 °C and ends at 549.03 °C with a sum loss of 88.51%. The endothermic peak appeared at the temperature of 375.86 °C. Similarly, a mass loss stage is observed on the TG curves of networks with a double-bond ratio of 2, as shown in Figure 3. The stage started from 339.15 °C and ended at 537.71 °C with a sum loss of 77.32%, and the endothermic peak is in the temperature of 374.01 °C. It indicates that the little change of thermal stabilities of the three types of samples may be due to double-bond ratio. The polymer networks with a double-bond ratio of 0.5 gets the most excellent thermal stability. From what has been discussed above, the thermal stability of the material was found to be significantly stronger than the PPF/PPF-DA networks with double-bond ratios (0.5, 1, 2), respectively [23].

Figure 5 shows the DSC curves of the polymer networks with double-bond ratios of 0.5, 1 and 2. The spectra show an exothermic reaction of the polymer by crosslinking. The onset curing temperature decreases from 343.90 to 279.00 °C by modifying the structure and double-bond ratios of the polymer. The three different double-bond-ratio materials showed glass transition temperature (Tg) at about 365.20, 352.40 and 344.60 °C, respectively. All three materials show a Tg value above 340 °C, which is higher than that of the PPF/PPF-DA networks [23]. Also, the enthalpy changes (ΔH) of the encapsulation materials are −34.21, −16.73 and −38.61 J/g, with double-bond ratios of 0.5, 1 and 2, respectively. The Tg of the samples became higher with decreasing double-bond ratio. This is because the propylene fumarate groups can enhance the rigidity of the polymer networks, which restrict the crosslinking. In other words, the double-bond ratio in polymer networks could react at the curing process, which decreases the crosslink density and the Tgs. This result was interpreted as the dependence of glass transition temperature on the chain flexibility, crosslinking density, and so on [35,36]. The high Tg value is essential for polymers to be used as emissive materials, indicating good potential use in encapsulation material.

### 3.4. Optical Properties

**Figure 6:**
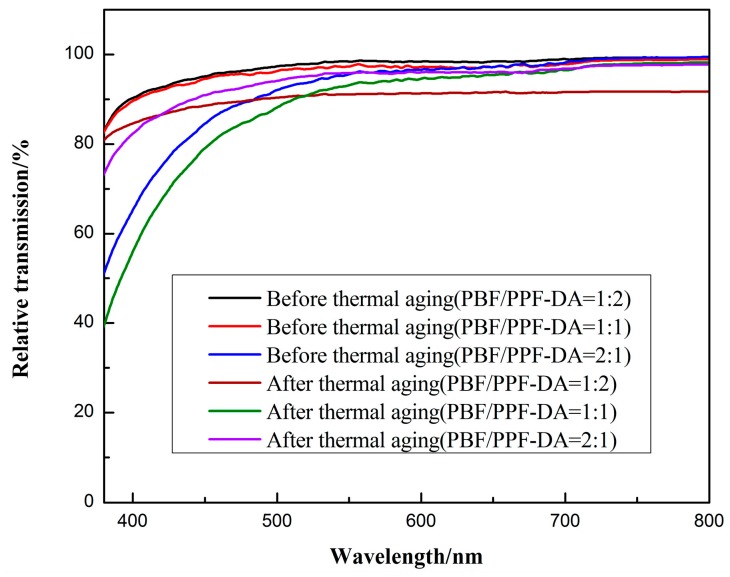
Transmittance of the prepared encapsulation material before and after thermal aging (one week).

Figure 6 exhibits the relationships between transmittance and wavelength for the prepared encapsulation material before/after thermal aging at 150 °C for one week. As shown in Figure 6, the UV-curable PBF/PPF-DA material has an optical transmittance of 99.64% before thermal aging. The transmittance is slightly higher than that previously reported in the literature [23]. In the visible light region, this material has high light transmittance even after thermal degradation, in order to be used in optical films. The network structure was therefore influenced by the amount of acrylate present in the reagent formulation, which increases with decreasing double-bond ratio. PBF/PPF-DA networks of double-bond ratio 1 show strong degradation after thermal aging, while the other two show relatively minimal effect. Increasing the PBF content within the reagent formulation results in a greater viscosity, which can limit the double-bond conversion by diffusion limitations [37]. 

**Figure 7:**
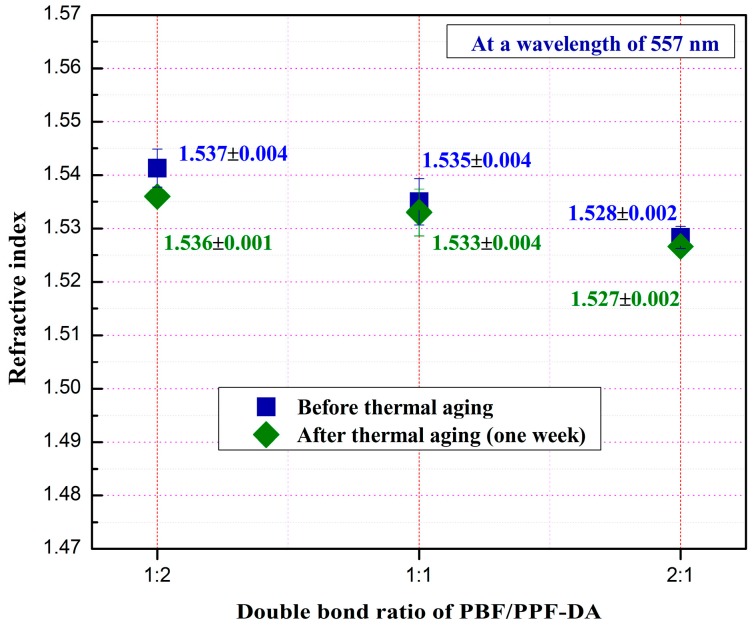
The refractive index of the prepared encapsulation material at a wavelength of 557 nm before and after thermal aging (one week).

The refractive index of an LED encapsulant is the main factor to develop high luminescence efficiency in an LED through enhancement of the light extraction efficiency. Figure 7 shows the refractive index for the prepared encapsulation material before and after thermal degradation aging. This encapsulation material has a higher refractive index (*n* = 1.537, 1.536) at a wavelength of 557 nm. Generally, the crosslinked polymer contains a higher refractive index functional group in comparison with its monomer.

### 3.5. Water Absorption Characterization of the Prepared Encapsulation Material

**Figure 8:**
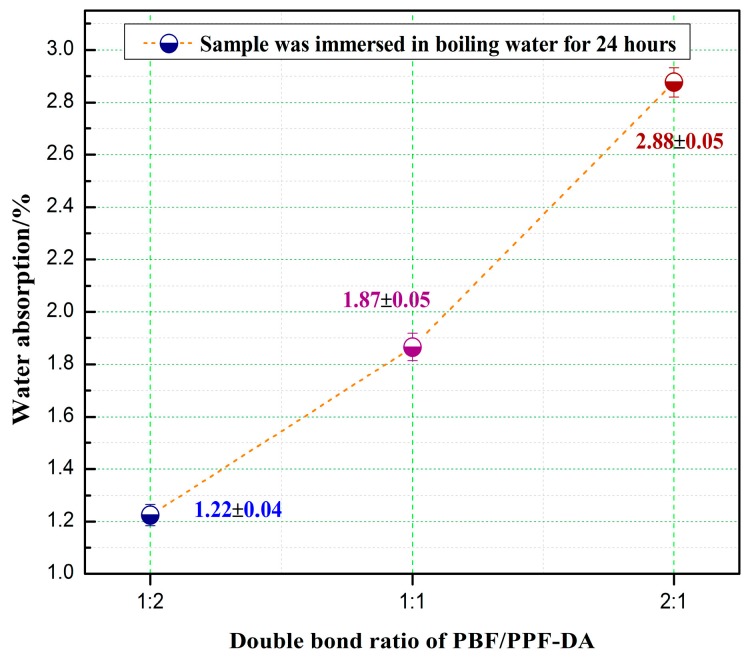
The water absorption ratio of the PBF/PPF-DA polymer networks with double-bond ratios 0.5, 1, and 2 (error bars represent mean ± SD for *n* = 3).

As shown in Figure 8, the water absorption ratios of the prepared encapsulation material were effectively improved by the chemical crosslinking of PBF and PPF-DA. With the increasing of double-bond ratios from 0.5 to 2, the water absorption ratios of the prepared encapsulation material were 1.22%, 1.87% and 2.88%, respectively. In comparison with other composite materials, the PBF-based polymer networks tested in this study shown much lower water absorption ratios [2]. The water absorption ratios of the prepared encapsulation material with a double-bond ratio of 2 (2.88%) was found to be significantly stronger than the networks with double-bond ratios of 0.5 (1.22%) and 1 (1.87%). It showed higher water absorption ratio and better hydrophilia because of the higher content of PBF in networks.

### 3.6. Mechanical Properties

**Figure 9:**
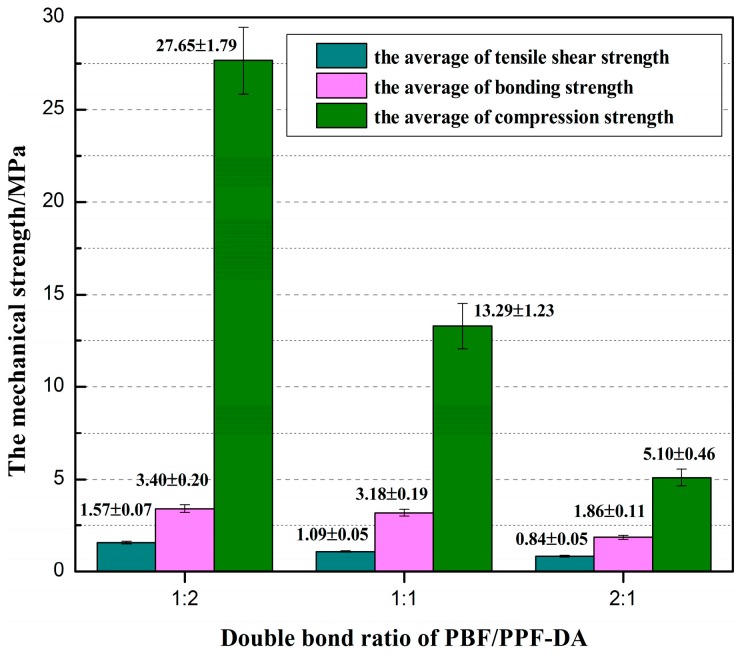
The tensile-shear, bonding, and compression strengths of the PBF/PPF-DA polymer networks with double-bond ratios 0.5, 1, and 2 (error bars represent mean ± SD for *n* = 5).

PBF/PPF-DA composite material has been shown to be a versatile material in which its mechanical properties can be altered by the initiator system or the crosslinking ratio [25]. The mechanical properties of the samples were examined in terms of their tensile-shear, bonding, and compression strengths, and the data are summarized in Figure 9. With the increasing of double-bond ratios from 0.5 to 2, the tensile-shear, bonding, and compression strengths of the prepared encapsulation materials were changed between 1.57~0.84, 3.40~1.56 and 27.65~5.10 MPa, respectively. Obviously, the bonding strengths were found to be stronger than that of the PPF/PPF-DA networks (2.39~1.09 MPa) [23]. The tensile-shear strength was 2 times more than that of PPF/PPF-DA networks with three types of double-bond ratios (0.79~0.38 MPa) [23]. These results demonstrate that the mechanical properties of PBF-based polymer networks can be tailored by varying the PBF/PPF-DA double-bond ratio. In comparison with other crosslinked PPF composites, the PBF-based polymer networks tested in this study showed improved mechanical properties [21,23,38].

Their tensile-shear strength testing results showed no significant difference that could be identified between the strength values with double-bond ratios of 1 (1.09 MPa) and 2 (0.84 MPa), but a more than 80% increase appeared in the strength values of PBF/PPF-DA polymer networks of double-bond ratio 0.5 (1.57 MPa) compared to 1 and 2. The bonding strength of the prepared encapsulation material with a double-bond ratio of 0.5 (3.40 MPa) was found to be significantly stronger than the networks with double-bond ratios of 1 (3.18 MPa) and 2 (1.86 MPa). The compression strength testing results showed a significant difference with double-bond ratios of 0.5, 1 and 2. The compression strength of the material with a double-bond ratio of 0.5 (27.65 MPa) was found to be significantly stronger than the networks with double-bond ratios of 1 (13.29 MPa) and 2 (5.10 MPa). It indicates that the mechanical properties of the PBF/PPF-DA polymer networks significantly decrease by increasing their double-bond ratio. For the BAPO initiator in an efficient dose range, the strengths of PBF/PPF-DA polymer networks depended on the number of acrylate bonds participating in the crosslinking reaction. From a viewpoint of mechanical property, this kind of PBF/PPF-DA network is suitable for the application of LED encapsulant [3,39].

## 4. Conclusions

In this study, a novel poly(butyl fumarate)-based LED encapsulation material was proposed. The encapsulation material was fabricated by UV-curing the newly synthesized PBF and PPF-DA. These polymers were successfully prepared from readily available starting compounds with a convenient procedure. The fabricated encapsulation material showed excellent optical transparency with high refractive index. Also, the good mechanical properties, lower water absorption, and excellent thermal stability were examined to confirm its feasibility for use as an encapsulant for LEDs. It is a most desirable combination for use in LED encapsulation, and would support the long lifetime and high efficiency of light extraction. This work has demonstrated strikingly effective properties of developments of polymers for high-performance LED encapsulants. In a word, current results indicate that PBF/PPF-DA polymer networks are potential encapsulating materials for light-emitting diode devices.
